# Mortality in nursing home residents stratified according to subtype of dementia: a longitudinal study over three years

**DOI:** 10.1186/s12877-022-02994-9

**Published:** 2022-04-05

**Authors:** Corinna Vossius, Sverre Bergh, Geir Selbæk, Jūratė Šaltytė Benth, Janne Myhre, Eivind Aakhus, Bjørn Lichtwarck

**Affiliations:** 1 The Research Centre for Age-related Functional Decline and Disease, Innlandet Hospital Trust, Ottestad, Norway; 2 Centre for Age-related Medicine, Stavanger University Hospital, Stavanger, Norway; 3 The Norwegian National Centre for Ageing and Health, Vestfold Hospital Trust, Tønsberg, Norway; 4 Department of Geriatric Medicine, University of Oslo, Oslo, Norway; 5 Institute of Clinical Medicine, University of Oslo, Oslo, Norway; 6 Health Services Research Unit, Akershus University Hospital, Oslo, Norway

**Keywords:** Nursing home, Mortality, Nursing home mortality, Dementia, Dementia subtypes, Long term nursing home residents

## Abstract

**Background:**

There are several subtypes of dementia caused by different pathophysiology and with different clinical characteristics. Irrespective subtype, the disease is progressive, eventually leading to the need for care and supervision on a 24/7 basis, often provided in nursing homes (NH). The progression rate and course of the disease might vary according to subtype. The aim of this study was to explore whether the mortality rate for NH residents varied according to the subtype of dementia.

**Methods:**

NH residents were followed from admission to NH over a period of 36 months or until death with annual follow-up examinations. Demographic and clinical data were collected. The diagnosis of dementia and its subtype at baseline (BL) were set according to international accepted criteria. Kaplan-Meier analysis was performed to estimate median survival time. A Cox regression model was estimated to assess the impact of dementia diagnosis and demographic and clinical variables on mortality.

**Results:**

A total of 1349 participants were included. When compared to persons with Alzheimer’s disease (AD), persons with frontotemporal dementia (FTD) and dementia with Lewy bodies or Parkinson’s disease dementia (DLB/PDD) were younger and had more neuropsychiatric symptoms. Median survival for the total sample was 2.3 years (95% confidence interval: 2.2–2.5). When compared to persons with AD, having no dementia or unspecified dementia was associated with higher mortality, while we found similar mortality in other subtypes of dementia. Higher age, male gender, poorer general health, higher dependency in activities of daily living, and more affective symptoms were associated with higher mortality.

**Conclusion:**

Mortality did not differ across the subtypes of dementia, except in persons with unspecified dementia or without dementia, where we found a higher mortality. With a median survival of 2.3 years, NH residents are in the last stage of their lives and care and medical follow-up should focus on a palliative approach. However, identifying the subtype of dementia might help carers to better understand and address neuropsychiatric symptoms and to customize medical treatment.

## Introduction

Dementia is a syndrome typically occurring in older adults and characterised by the loss of memory and other cognitive abilities, functional impairment, and behavioural symptoms. In Norway, the prevalence of dementia has been estimated to 100,000 in 2020 or 1.8% of the general Norwegian population, and the number is projected to rise to more than 236,000, respectively 2.6% of the population by 2050 [1]. There are several subtypes of dementia caused by different pathophysiology and with different clinical characteristics. The most common form in nursing homes (NH) is Alzheimer’s disease (AD), with about 70% of cases, followed by vascular dementia (VaD; 8%), dementia with Lewy bodies or Parkinson’s disease dementia (DLB/PDD; 8%), and frontotemporal dementia (FTD; 4%) [2]. Irrespective of subtype, the disease is progressive, eventually leading to the need for care and supervision on a 24/7 basis. However, previous research has shown that the progression rate and course of the disease might vary according to the subtype, with persons with DLB/PDD having a shorter interval between diagnosis and NH admission and a shorter survival [3–6].

In Norway, the highest level of formal care is offered in NH. Most formal care is organised and financed by the municipalities. The necessity for NH admission is evaluated by the municipal health and social services according to functional impairment of the patient. About 50% of persons with dementia have been admitted to NH within three years after the diagnosis of dementia is made, and 84% of NH residents have dementia [7–9].

Population projections predict an increase in the elderly population in the whole world and hence an increase in the prevalence of persons with dementia, leading again to an increased need for NH services [1, 7]. Thus, knowledge about the time from NH admission to death and predictors for mortality are relevant parameters when dimensioning for future care facilities and planning for evidence-based end of life care for NH residents. Previous research found that mean survival time for nursing home residents was just over two years in both Norwegian and Danish studies [7, 10, 11]. Predicting factors for mortality were higher age, male gender, low functioning in activities of daily living (ADL), poorer physical health, low nutrition status, and more severe dementia [11–15]. A previous study showed that mortality varied across the subtypes of dementia in persons included at the point of diagnostic workup, with persons with unspecified dementia having the highest survival, followed by AD and mixed dementia (VaD/AD), while persons with VaD and DLB/PDD had the shortest survival [16]. The aim of this study was to explore whether the subtype of dementia was a factor predicting mortality as well in long-term NH residents.

## Material and methods

### Settings and participants

In this study we combined the data from two clinical studies following NH residents from admission and over the whole course of their NH stay with regular follow-up (FU) examinations. Both studies recruited participants as convenience sample from several counties in Norway, including both rural and urban areas. The demographic and clinical characteristics from both cohorts were similar. These studies were:

- Resource Use and Disease Course in Dementia - Nursing Home (REDIC-NH) including 696 persons. Participants were followed over a period of three years or until death, with clinical examinations at baseline (BL) and every six months thereafter. The study took place between 2012 and 2017 [17].
- Cooperation between The Department of Old Age Psychiatry, Innlandet Hospital Trust, and municipal nursing homes in the Innlandet County (SAM-AKS III). SAM-AKS III is an ongoing study that started in 2014. For this study we included 797 residents with a minimum of three years follow-up that were included in the study from January 2014 to December 2017. Participants were followed with clinical examination at BL and yearly FU afterwards [18].

Inclusion criteria were: (i) 65 years of age or older in REDIC-NH and 60 years of age or older in SAM-AKS III or (ii) having dementia irrespective of age. (iii) In addition, expected survival should be six weeks or more for REDIC-NH and four weeks or more for SAM-AKS III. Only residents that completed BL assessment were included in the study. BL assessment was aimed to be completed within four weeks after inclusion, but the mean interval between admission and the completed BL assessment was 13.1 weeks (Standard deviation (SD) 9.2). A total of 3484 persons were eligible for study inclusion, whereof 1991 (58%) did not participate because they or their next of kin did not wish to consent (27%); the resident died before BL assessment (18%); or other reasons (55%). Those not included were younger (83.7 vs 84.7 years; *p* < 0.001) and more often male than those included (41 vs 35%; *p* < 0.001).

For the present study we applied the following exclusion criteria: (i) Participants, where the exact date of NH admission or end of observation period could not be established, (ii) participants who moved back home during the observation period, and (iii) cases where the interval between NH admission and BL examination was more than one year.

Participants were followed over a period of 36 months from the time of NH admission or until death. For this study we included data from the clinical monitoring after one year (FU1), two years (FU2) and three years (FU3). Due to delays in follow-up assessments, FU3 might have taken place after the end of the observation period.

### Data collection

Data collection was performed by trained healthcare workers at the NH, mainly registered nurses, under supervision of research nurses. The research nurses completed a five-day training prior to study start, while the data collectors completed a two-day training. Data were collected through structured interviews with the patient and a caregiver [17].

All rating scales and inventories were applied using validated Norwegian versions. The following demographic and clinical data were collected:

Demographic data, including gender, age, and living status before admission to NH, were collected by reviewing the patient’s journal.

Diagnoses of dementia and subtype of dementia at BL were set according to internationally recognised criteria. The diagnosis of dementia was set independently by four of the authors (SB, BL, GS, EA). Three were specialists in psychiatry and one was general practitioner and nursing home physician, and all were experienced in old age psychiatry and research. Diagnoses were based on all available information about the participants. Each case was reviewed by two of the physicians, and if no consensus was reached, a third psychiatrist was consulted. Dementia, Alzheimer’s disease, vascular dementia, and mixed AD/VaD were diagnosed according to the ICD-10 criteria [19]; DLB/PDD was diagnosed according to the DLB consortium criteria [20]. Frontotemporal dementia was diagnosed according to the Manchester-Lund criteria [21].

The Clinical Dementia Rating Scale (CDR) was applied to assess the severity of dementia. The rating scale comprises six items [22], where total CDR score is given based on an algorithm. For statistical purposes we calculated the CDR-sum of boxes (CDR-SoB) that offers an extended range of values compared to the algorithm-based scoring, and is calculated by adding the item scores (range 0–18), where higher scores indicate more severe dementia [23].

The Neuropsychiatric Inventory (NPI) assesses neuropsychiatric symptoms. The instrument contains 12 items and is conducted as an interview with a caregiver. Severity (scored 0–3) was multiplied by frequency (scored 0–4), giving an item score from 0–12, where higher scores indicate more severe symptoms [24, 25]. Based on a previous principal component analysis, we created the following sub-syndromes: NPI-Agitation (agitation/aggression, disinhibition, and irritability), NPI-Psychosis (delusions and hallucinations), and NPI-Affective (depression and anxiety) [17].

Physical Self-Maintenance Scale (PSMS) consists of six items (scored 1–5) and assesses personal activities of daily living (PADL) function. The overall score ranges from 6 to 30, where higher scores indicate higher PADL dependency [26].

General Medical Health Rating (GMHR) rates physical health. It consists of one item, with the four categories excellent, good, moderate, or poor [27].

Body mass index (BMI) relates a person’s weight to the height (BMI = weight/height^2^).

### Ethics

The residents’ capacity to consent to participation in the study was considered by the NH staff, including the physician. Written informed consent was obtained by the participants with full capacity to consent, or by next-of-kin on behalf of the participants in case of reduced capacity to consent. The Regional Ethics Committee for Medical research in South-Eastern Norway approved of the studies (2011/1378a and 2014/917) [11].

### Statistics

Demographic factors and clinical symptoms were described by means and standard deviations (SDs) or frequencies and percentages. The group differences were analysed by Student’s t-test for continuous variables and χ^2^-test for categorical variables. To evaluate demographic and clinical differences across the subtypes of dementia, we compared the various subtypes to AD as the largest subtype. Missing values for PSMS, CDR, and NPI items were imputed for cases with fewer than 50% missing among all items by generating an empirical distribution based on non-missing cases for each item and drawing a random number from it. Kaplan-Meier analysis was performed to estimate to assess median survival time across the subtypes of dementia. An extended Cox regression model was estimated to assess the impact of dementia diagnosis, demographic variables, and time-dependent clinical variables on mortality. Bivariate and multiple models were estimated. The model assumptions were assessed by standard statistical tests. A sensitivity analysis was performed by including only participants with BL assessment within 90 days to explore the impact of the time interval between NH admission and BL assessment on the distribution of subtypes of dementia and median survival time. Results with *p*-values below 0.05 were considered statistically significant. The analyses were performed in SPSS v26 and SAS v9.4.

## Results

### Study cohort

A total of 1493 persons participated in the study at BL examination (696 from REDIC-NH and 797 from SAM-AKS III). Of these, a total of 144 were excluded from further analysis (18 due to missing date of NH admission or end of observation period; 37 due to an interval of more than one year between admission and BL examination; 60 moved back home; 26 withdrew consent; and in three participants the reason for study termination could not be established). Those excluded where younger than those included (mean age 82.6 vs 84.7 years, *p* = 0.002) and a lower proportion had dementia (72% vs 84%, *p* = 0.001).

**Fig. 1:**
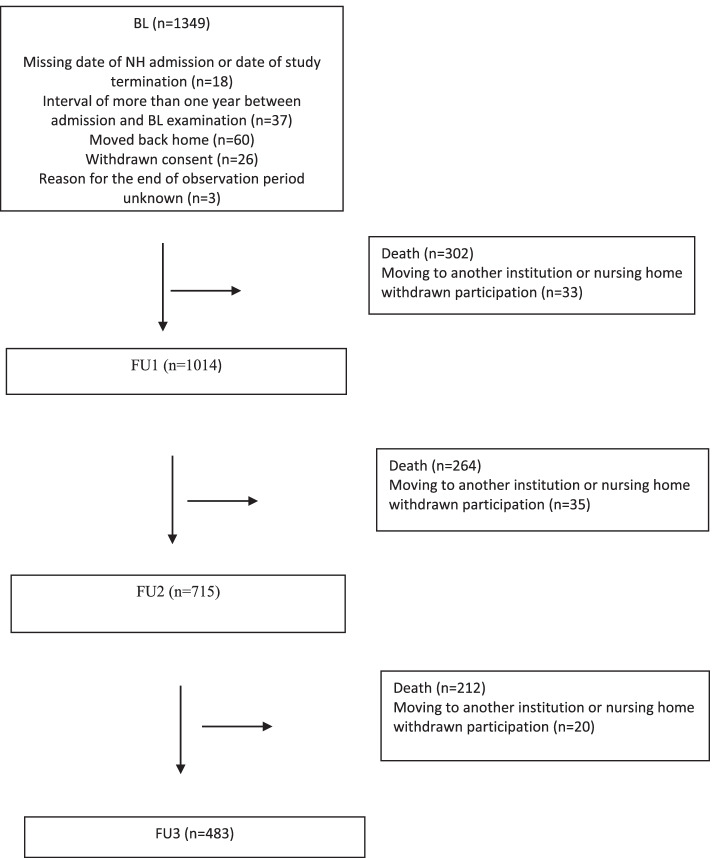
Flow chart of the included participants and attrition during the study period. BL = baseline, FU = follow up

Figure 1 shows a flow chart of the included participants and attrition during the study period.

Table 1 shows demographic and clinical characteristics throughout the observation period and separately BL characteristics for those deceased during the observation period, while Table 2 shows demographic and clinical characteristics for the subtypes of dementia at BL. When compared to persons with AD, persons with FTD and DLB/PDD were younger (*p* = 0.011 and *p* < 0.001, respectively) and had more neuropsychiatric symptoms (*p* = 0.003 and *p* = 0.001). Persons with FTD had more agitation (*p* < 0.001), and persons with DLB/PDD more psychosis (*p* < 0.001). Persons with an unspecified dementia had more affective symptoms (*p* = 0.040). Persons with DLB/PDD lived more rarely alone before NH admission (*p* = 0.002) and had a higher PADL-dependency at BL (*p* < 0.001).

**Table 1:** Demographic and clinical characteristics throughout the observation period and BL characteristics for those deceased during the observation period

	BL	FU1	FU2	FU3	Deceased during study period, BL characteristics
Participants (%)	1349	1014 (75.1)	715 (53.0)	483 (35.8)	778 (57.7)
Examination carried out, n (%)	1349 (100)	849 (84)	558 (78)	339 (70)	
Difference since study start, n (%)					
- Deceased	–	302 (22.4)	566 (42.0)	778 (57.7)	–
- other^a^		33 (2)	68 (4)	88 (6)	
Difference since year before, n (%)					
- Deceased	–	302 (22.4)	264 (26.0)	212 (29.7)	–
- other		33 (2)	35 (2)	20 (1)	
Gender, female (%)	874 (65)	677 (67)	485 (68)	337 (70)	482 (62)
Age, mean (SD)	84.7 (7.5)	84.5 (7.5)	83.7 (7.7)	83.5 (7.7)	85.8 (7.0)
Living alone before NH; n (%)	937 (69)	714 (71)	506 (71)	340 (71)	541 (70)
GMHR poor or moderate, n (%)	648 (52)	481 (58)	344 (63)	205 (65)	414 (58)
BMI, mean (SD)	24.3 (4.9)	25.2 (6.6)	25.4 (5.3)	25.2 (4.7)	23.8 (5.0)
PSMS, mean (SD)	15.0 (4.9)	16.5 (4.8)	18.1 (4.9)	19.5 (5.0)	15.9 (4.5)
NPI, mean (SD)	13.8 (16.8)	15.5 (17.3)	18.5 (20.0)	18.1 (19.6)	14.4 (17.0)
NPI-AGI, mean (SD)	4.3 (7.2)	5.3 (7.8)	6.7 (8.9)	6.4 (8.3)	4.4 (7.0)
NPI_PSY, mean (SD)	1.7 (3.7)	2.0 (3.9)	2.5 (4.3)	2.5 (4.5)	1.8 (3.8)
NPI-AFF, mean (SD)	3.7 (4.3)	3.4 (5.2)	3.6 (5.3)	3.5 (5.5)	3.8 (4.4)
CDR-SoB	10.3 (4.3)	11.9 (4.1)	13.1 (4.1)	13.8 (4.0)	10.5 (4.4)
Type of dementia, n (%)					
- No dementia	204 (15.1)	127 (12.5)	82 (11.5)	62 (12.8)	126 (16.3)
- AD	766 (57.0)	605 (59.7)	422 (59.0)	284 (58.8)	434 (55.8)
- VaD	81 (6.0)	68 (6.7)	49 (6.9)	33 (6.8)	42 (5.4)
- AD/VaD	64 (4.7)	47 (4.6)	35 (5.0)	25 (5.2)	36 (4.6)
- FTD	99 (7.3)	74 (7.3)	59 (8.3)	36 (7.5)	57 (7.3)
- DLB/PDD	66 (4.9)	52 (5.1)	38 (5.3)	25 (5.2)	39 (5.0)
- Unspecified	58 (4.3)	31 (3.1)	21 (2.9)	12 (2.5)	39 (5.0)
- Cannot be evaluated	11 (0.8)	10 (1.0)	8 (1.1)	6 (1.2)	5 (0.7)

*BL* baseline, *FU* follow-up, *SD* standard deviation, *GMHR* General medical health rating, *BMI* Body mass index, *PSMS* Physical self-maintenance scale, *NPI* Neuropsychiatric inventory, *NPI-AGI* NPI sub-syndromes agitation/aggression, disinhibition and irritability, *NPI-PSY* NPI sub-syndromes delusions and hallucinations, *NPI-AFF* NPI sub-syndromes depression and anxiety, *CDR-SoB* Clinical dementia rating scale – sum of boxes, *AD* Alzheimer’s disease, *VaD* vascular dementia, *FTD* frontotemporal dementia, *DLB* dementia with Lewy bodies, *PDD* Parkinson’s disease dementia

^a^Other = participants moved to another institution or nursing home has withdrawn from participation

**Table 2:** Demographic and clinical characteristics for the subtypes of dementia at BL, mortality at the end of the observation period, median survival time and a sensitivity analysis including only participants with the BL assessment within 90 days after NH admission

	All	No demen-tia	AD	VaD	AD/VaD	FTD	DLB/PDD	Un-specified dementia
N (%)	1349	204 (15.1)	766 (57.0)	81 (6.0)	64 (4.7)	99 (7.3)	66 (4.9)	58 (4.3)
Age, mean (SD)	84.7 (7.5)	86.7 (7.7)	84.8 (7.3)	83.5 (7.0)	85.2 (7.9)	82.7 (8.0)	81.2 (7.9)	84.4 (6.4)
Gender female (%)	874 (65)	133 (65)	520 (68)	52 (64)	39 (61)	67 (68)	23 (35)	34 (60)
Living alone before NH; n (%)	937 (69)	155 (76)	530 (70)	59 (73)	44 (69)	62 (64)	34 (52)	45 (78)
GMHR poor or moderate, n (%)	648 (52)	104 (51)	343 (48)	51 (63)	32 (55)	48 (51)	40 (64)	25 (53)
BMI, mean (SD)	24.3 (4.9)	25.0 (5.9)	24.1 (4.8)	24.8 (5.0)	24.4 (4.7)	24.5 (4.2)	23.9 (4.9)	24.7 (5.0)
PSMS, mean (SD)	15.0 (4.9)	14.7 (4.5)	14.8 (4.3)	16.0 (4.4)	14.9 (4.6)	15.0 (4.9)	16.9 (4.4)	16.4 (4.2)
NPI, mean (SD)	13.8 (16.8)	6.8 (10.5)	14.0 (16.7)	13.0 (15.6)	14.8 (17.7)	19.7 (18.0)	21.4 (21.6)	18.4 (19.1)
NPI-AGI, mean (SD)	4.3 (7.2)	1.4 (3.4)	4.4 (7.1)	4.1 (7.3)	5.0 (7.2)	7.9 (8.7)	6.2 (9.2)	5.2 (8.1)
NPI_PSY, mean (SD)	1.7 (3.7)	0.6 (2.1)	1.6 (3.5)	1.1 (2.8)	2.8 (5.1)	2.4 (4.5)	4.4 (5.6)	1.1 (3.2)
NPI-AFF, mean (SD)	3.7 (4.3)	2.5 (4.7)	3.9 (5.7)	3.3 (5.0)	3.0 (5.1)	4.2 (5.7)	4.0 (6.0)	5.6 (6.6)
CDR-SoB, mean (SD)	10.3 (4.3)	5.0 (3.8)	11.3 (3.5)	11.1 (3.4)	10.5 (3.8)	11.9 (3.3)	11.4 (3.6)	10.9 (4.3)
Deceased during observation period, n (%)	778 (57.7)	126 (61.8)	434 (57.0)	42 (51.9)	36 (56.3)	57 (57.6)	39 (59.1)	39 (67.2)
Median survival (CI)	2.3 (2.2–2.5)	1.6 (1.1–2.0)	2.4 (2.2–2.7)	2.6 (−)	2.5 (2.1–3.0)	2.7 (2.4–2.9)	2.6 (1.8–3.4)	1.4 (0.6–2.2)
Sensitivity analysis: Participants with BL assessment within 90 days after NH admission								
N (%)	795	119 (15.0)	471 (59.2)	40 (5.0)	34 (4.3)	51 (6.4)	42 (5.3)	31 (3.9)
Median survival (CI)	2.3 (2.0–2.5)	1.4 (0.9–1.8)	2.3 (1.9–2.6)	2.6 (1.9–3.3)	2.9 (−)	2.7 (2.2–3.2)	2.7 (1.8–3.5)	1.2 (0.6–1.8)

*BL* Baseline, *NH* Nursing home, *AD* Alzheimer’s disease, *VaD* vascular dementia, *FTD* frontotemporal dementia, *DLB* dementia with Lewy bodies, *PDD* Parkinson’s disease dementia, *SD* standard deviation, *NH* nursing home, *GMHR* General medical health rating, *BMI* Body mass index, *PSMS* Physical self-maintenance scale, *NPI* Neuropsychiatric inventory, *NPI-AGI* NPI sub-syndromes agitation/aggression, disinhibition and irritability, *NPI-PSY* NPI sub-syndromes delusions and hallucinations, *NPI-AFF* NPI sub-syndromes depression and anxiety, *CDR-SoB* Clinical dementia rating scale – sum of boxes, *CI* Confidence interval

### Mortality

At the end of the study period, 57.7% of the participants where deceased and 6.5% were lost to follow up due to moving to another institution or the nursing home withdrawing from participation. Median survival for the whole cohort was 2.3 years (95% confidence interval (CI) 2.2–2.5). Table 1 displays BL characteristics for those deceased during the observation period. When compared to participants still alive after 36 months, those who deceased were older (83.2 vs 85.8 years; *p* < 0.001), more likely men (31% vs 38%; *p* = 0.009), had more often poor or moderate general health (44% vs 58%; *p* < 0.001), were more dependent in PADL (13.9 vs 15.9; *p* < 0.001), and had a more severe degree of dementia (10.0 vs 10.5; *p* = 0.024).

Table 2 shows median survival stratified according to subtypes of dementia. NH residents with no dementia had a significantly shorter survival than persons with AD with 1.6 vs 2.4 years (95% CI 1.1–2.0). Persons with unspecified dementia had a median survival of 1.4 years (95% CI 0.6–2.2), but the result was not statistically significant different from persons with AD. Table 3 shows the results of the bivariate Cox regression model and the multiple model. When compared to persons with AD, having no dementia or unspecified dementia was associated with higher mortality. Of the demographic and clinical characteristics, higher age, male gender, poorer general health, higher PADL-dependency, and more affective symptoms were associated with higher mortality.

**Table 3:** Results of the cox model

Covariates	Bivariate models		Multiple models	
	HR (95% CI)	*p*-value	HR (95% CI)	*p*-value
Dementia diagnosis				
No dementia	1.36 (1.07; 1.71)	**0.011**	1.44 (1.08; 1.92)	**0.012**
AD	1		1	
VaD	0.85 (0.58; 1.25)	0.416	0.81 (0.55; 1.20)	0.293
AD/VaD	0.97 (0.64; 1.47)	0.887	0.91 (0.60; 1.38)	0.648
FTD	1.08 (0.79; 1.48)	0.639	1.18 (0.86; 1.62)	0.305
DLB/PDD	0.92 (0.61; 1.39)	0.680	0.74 (0.49; 1.13)	0.168
Unspecified dementia	1.79 (1.19; 2.70)	**0.005**	1.76 (1.17; 2.67)	**0.007**
Patient characteristics				
Age at admission	1.03 (1.02; 1.05)	**<0.001**	1.04 (1.03; 1.05)	**<0.00**1
Gender, male	1.24 (1.05; 1.47)	**0.014**	1.32 (1.10; 1.58)	**0.003**
Lived alone before admission, no	1.12 (0.94; 1.34)	0.202	1.09 (0.90; 1.33)	0.360
Time dependent variables				
GMHR, good or excellent	0.64 (0.54; 0.76)	**<0.001**	0.80 (0.67; 0.96)	**0.018**
PSMS	1.10 (1.08; 1.12)	**<0.001**	1.10 (1.08; 1.13)	**<0.001**
NPI-AGI	1.00 (0.99; 1.01)	0.905	0.99 (0.97; 1.00)	0.056
NPI- PSY	1.02 (1.00; 1.04)	0.142	1.02 (1.00; 1.04)	0.234
NPI-AFF	1.02 (1.00; 1.03)	**0.026**	1.02 (1.00; 1.04)	**0.022**
CDR-SoB	1.03 (1.01; 1.06)	**0.002**	1.00 (0.97; 1.03)	0.891

*HR* Hazard ratio, *CI* Confidence interval, *AD* Alzheimer’s disease, *VaD* vascular dementia, *FTD* frontotemporal dementia, *DLB* dementia with Lewy bodies, *PD* Parkinson’s disease dementia, *GMHR* General medical health rating, *PSMS* Physical self-maintenance scale, *NPI* Neuropsychiatric inventory, *NPI-AGI* NPI sub-syndromes agitation/aggression, disinhibition and irritability, *NPI-PSY* NPI sub-syndromes delusions and hallucinations, *NPI-AFF* NPI sub-syndromes depression and anxiety, *CDR-SoB* Clinical dementia rating scale – sum of boxes, *CI* Confidence interval

A sensitivity analysis including only those study participants that completed BL assessment within 90 days after NH admission showed that the distribution of the subtypes of dementia and the median survival times were comparable to the findings for the whole cohort. However, in the Cox regression model higher mortality was only associated with higher age, poorer general health, and higher PADL-dependency.

**Fig. 2:**
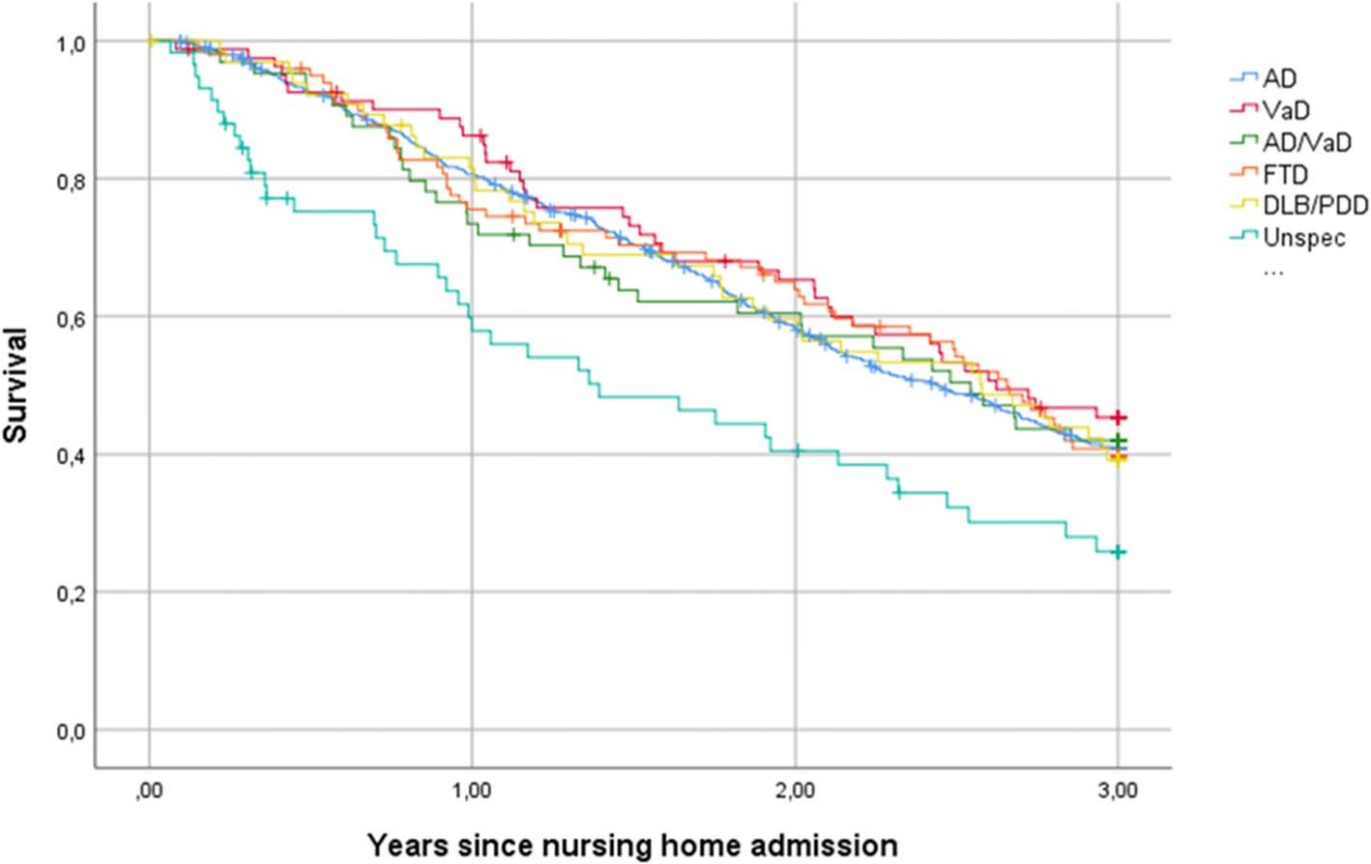
Kaplan-Meier survival functions for the subtypes of dementia. AD = Alzheimer’s disease: VaD = vascular dementia; FTD = frontotemporal dementia; DLB = dementia with Lewy bodies; PDD=Parkinson’s disease dementia; Unspec = unspecified dementia

Figure 2 displays Kaplan-Meier survival functions for the various subtypes of dementia. We found that the slopes of the curves were more or less parallel with an even decline over the course of three years, with only the curve for unspecified dementia declining faster.

## Discussion

We evaluated the time of survival for the different subtypes of dementia in a cohort of 1349 long-term NH residents included at admission. Median survival was 2.3 years for the whole cohort. Residents with a shorter time of survival were those without dementia (median 1.6 years) and those with an unspecified dementia (median 1.4 years, not statistically significantly different), while the different subtypes of dementia did not show any significant difference in survival. Additional predictors of higher mortality were higher age, male gender, poorer general health, higher PADL-dependency, and more affective symptoms. Our findings of a median survival of 2.3 years, and the demographic and clinical factors associated with higher mortality, are consistent with previous research [12–15].

Previous research has shown that overall survival was shortest in persons with VaD and DLB/PDD and that persons with DLB/PDD were admitted to NH earlier after the diagnosis of dementia than persons with AD [3, 16]. We found that age and the level of neuropsychiatric symptoms were the BL variables showing the greatest variance across the subtypes of dementia, with persons with DLB/PDD and FTD being younger and having more neuropsychiatric symptoms. These findings are in line with previous research reporting that neuropsychiatric symptoms are associated with the caregiver burden, while caregiver burden again is associated with the time to NH admission [28, 29]. Thus, persons with DLB/PDD and FTD were admitted to NH at a younger age, possibly partly due to more severe neuropsychiatric symptoms, but once admitted to NH the survival time did not differ from other subtypes of dementia.

The shorter survival time in persons without dementia might be due to higher morbidity as previous research has shown that nursing home residents without dementia have a higher frequency of physical diagnoses than those with dementia [30]. We also found that persons with unspecified dementia had a shorter survival time than other subtypes of dementia. This is in contrary to previous research that evaluated the survival for various aetiologies of dementia and found that DLB/PDD had the shortest survival while persons with unspecified dementia had the longest survival time [16]. The term “unspecified dementia” subsumes individuals that fulfil the diagnostic criteria for dementia, but where the subtype of dementia cannot be determined. Thus, this sub-cohort might be a quite heterogeneous group, where some individuals have developed severe neuropsychiatric symptoms that render an exact diagnosis difficult, while other participants might be admitted to NH due to physical diseases as their main health challenge, and the cognitive impairment in these cases might be just a comorbidity or a concomitant delirium.

Except for unspecified dementia, we did not find any differences in survival across the subtypes of dementia. A median survival of 2.3 years indicates that nursing home residents in general are in the last phase of their lives, where care and medical follow-up should take a palliative approach, focussing on the alleviation of distressing symptoms and on good quality of life rather than life-prolonging measures [31]. However, an exact diagnosis of the subtype of dementia will help both the next of kin and the NH staff to better understand and address neuropsychiatric symptoms. In addition, it might give useful information for the choice of medical treatment with for example anticholinergics or neuroleptics.

### Strengths and limitations of the study

We followed a large cohort of 1349 participants in a longitudinal design over three years, with clinical examinations every year. High quality of the data collection was secured by a standardized interview carried out by healthcare workers with adequate training under the supervision of research nurses. Furthermore, the Norwegian health and social system provides a rather homogenous environment for health service research as there are hardly any private actors on the market. Institutional care is provided by the municipalities with comparable criteria for NH admission and standards of care and medical follow up during NH residency [11]. Even if the inclusion period for the study lasted from 2011 to 2017, we could show a stability of the demographic and clinical characteristics over time. In addition, there have not been any relevant changes in the Norwegian public health sector that would indicate major changes in the NH population, thus ensuring that our finding will still be relevant for today’s NH population.

Still, the main weakness of this study is that our sample might not be representative of the general NH population in Norway. Only participants that completed the BL examination were included, and mean time from admission to BL was 13.1 weeks. Thus, 338 persons died before BL examination, representing 10% of all residents eligible for the study. Therefore, the overall survival time might be overestimated, and we have no information if some subtypes of dementia might be overrepresented among those dying shortly after NH admission. However, the sensitivity analysis that explored the impact of the interval between NH-admission to BL assessment showed both a comparable distribution of the subtypes of dementia and comparable median survival times. In the Cox regression analysis, the difference in survival between the subtypes of dementia was no longer significant, possibly due to reduced statistical power. More than half of the eligible residents for this study did not participate. These were more often male and at mean one year younger than the study cohort, indicating a selection bias while recruiting the study participants. The subtype of dementia was determined based on the data collected at NH admission and thus rather late in the course of the disease, when distinguishing between subtypes of dementia can be more challenging. Even if the dataset included information about the first symptoms of the dementia as reported by the next of kin and the diagnosis was set by experienced physicians and according to the guidelines, this may have led to mis-classification in some cases. In addition, the collected data is not sufficient to explain the higher mortality rates in persons without dementia and with unspecified dementia.

## Conclusion

Mortality did not differ across the subtypes of dementia, except for in persons with unspecified dementia or without dementia, where we found a higher mortality. With a median survival of 2.3 years, NH residents are in the last stage of their lives and care and medical follow-up should focus on a palliative approach, whenever this is according to the resident’s wish. However, identifying the subtype of dementia might help carers to better understand and address neuropsychiatric symptoms and to customize medical treatment.

## Data Availability

The datasets generated and analysed during the current study are not publicly available as public availability was not consented to by the study participants and not approved by the Ethics Committee. Data is available from the corresponding author on reasonable request.

## Abbreviations

NH: Nursing home
BL: Baseline
AD: Alzheimer’s disease
FTD: Frontotemporal dementia
DLB: Dementia with Lewy bodies
PDD: Parkinson’s disease dementia
VaD: Vascular dementia
ADL: Activities of daily living
FU: Follow-up
REDIC-NH: Resource Use and Disease Course in Dementia - Nursing Home
SAM-AKS III: Cooperation between The Department of Old Age Psychiatry, Innlandet Hospital Trust, and municipal nursing homes in the Innlandet County.
SD: Standard deviation
CDR: Clinical Dementia Rating Scale
CDR-SoB: CDR-sum of boxes
NPI: Neuropsychiatric Inventory
NPI-AGI: NPI-Agitation
NPI-PSY: NPI-Psychosis
NPI-AFF: NPI-Affective
PSMS: Physical Self-Maintenance Scale
GMHR: General Medical Health Rating
BMI: Body mass index
